# Chitosan–Orientin Nanoparticles Attenuate Hypoxia-Induced Pulmonary Inflammation in Rats

**DOI:** 10.3390/biom16091306

**Published:** 2026-09-09

**Authors:** Gülfem Özduygu, İhsan Topaloğlu, Çağrı Atasoy, Eren Erdoğdu, Güntuğ Batıhan, Semih Çiftçi, Volkan Gelen, Kübra Kaya, Adem Kara, Ali Yeşildağ, Elif Erbaş, Deniz Ekinci

**Affiliations:** 1 Department of Pulmonology, Faculty of Medicine, Istanbul University, Istanbul 34093, Turkey; 2 Department of Pulmonology, Faculty of Medicine, Kafkas University, Kars 36000, Turkey; ras-topal@hotmail.com (İ.T.); cagriatasoy92@gmail.com (Ç.A.); sc.semhcftc@gmail.com (S.Ç.); 3 Department of Thoracic Surgery, Faculty of Medicine, Istanbul University, Istanbul 34093, Turkey; eren.erdogdu@istanbul.edu.tr; 4 Department of Thoracic Surgery, Faculty of Medicine, Canakkale Onsekiz Mart University, Canakkale 14020, Turkey; gbatihan@hotmail.com; 5 Department of Physiology, Veterinary Faculty, Kafkas University, Kars 36000, Turkey; gelen_volkan@hotmail.com (V.G.); kubrakaya1306@gmail.com (K.K.); 6 Department of Genetics, Faculty of Science, Erzurum Technical University, Erzurum 25000, Turkey; adem.kara@erzurum.edu.tr; 7 Department of Bioengineering, Faculty of Engineering and Architecture, Kafkas University, Kars 36000, Turkey; aliyesildag@kafkas.edu.tr; 8 Department of Histology and Embryology, Veterinary Faculty, Atatürk University, Erzurum 25000, Turkey; eliferbas@atauni.edu.tr; 9 Department of Agricultural Biotechnology, Faculty of Agriculture, Ondokuz Mayıs University, Samsun 25000, Turkey; deniz.ekinci@omu.edu.tr

**Keywords:** chitosan nanoparticles, hypoxia-induced lung injury, pulmonary inflammation, orientin, oxidative stress

## Abstract

**Background:** Hypoxia triggers pulmonary inflammation and oxidative stress, leading to endothelial dysfunction, apoptosis and lung injury. Although the flavonoid orientin exhibits potent antioxidant and anti-inflammatory properties, its pharmacokinetic behavior remains incompletely characterized. Nanoparticle-based delivery systems may enhance efficacy. This study investigated whether Orientin delivered via chitosan nanoparticles (CNP–orientin) protects against hypoxia-induced pulmonary inflammation, oxidative stress, and tissue injury in rats. **Methods:** A total of 48 female Sprague–Dawley rats were randomized into four normoxic and four hypoxic groups (*n* = 6 per group). Rats in the hypoxic groups were exposed to intermittent hypoxia (7% O_2_, 8 h/day for 7 days). Hypoxic rats were allocated to experimental groups, including an untreated hypoxia group and groups receiving orientin alone, chitosan nanoparticles alone or orientin-loaded chitosan nanoparticles (CNP–orientin), while normoxic rats served as controls. Hypoxic exposure was conducted using a controlled glove box system. At the end of the protocol, blood samples were collected for serum inflammatory marker analysis, and lung tissues were harvested to assess oxidative stress parameters, hypoxia- and inflammation-related signaling pathways, apoptosis-related gene expression, and histopathological lung injury and fibrosis. **Results:** Intermittent hypoxia significantly increased pulmonary *HIF-1α and iNOS* expression, oxidative stress markers (increased malondialdehyde and decreased superoxide dismutase and glutathione), pro-inflammatory cytokines (TNF-α, IL-1β), apoptotic signaling and histopathological lung injury compared with normoxic controls (*p* < 0.05). Treatment with orientin or chitosan nanoparticles alone attenuated hypoxia-associated biochemical and molecular alterations compared to untreated hypoxic rats (*p* < 0.05). Notably, CNP–orientin treatment significantly attenuated multiple oxidative stress, inflammatory, and apoptosis-related alterations compared with untreated hypoxic rats (*p* < 0.05). These protective effects were observed despite the substantially lower orientin-equivalent dose in the CNP–orientin formulation compared with free orientin. NF-κB and TNF-α expression, caspase-3 levels, antioxidant markers, and Bcl-2 expression differed significantly among hypoxic groups (*p* < 0.05). Histopathological lung injury and Modified Ashcroft fibrosis scores were significantly lower in the CNP–orientin group than in the untreated hypoxic group (*p* < 0.01 and *p* < 0.001, respectively). **Conclusions:** Orientin-loaded chitosan nanoparticles effectively mitigate hypoxia-induced pulmonary inflammation, oxidative stress, apoptosis and fibrotic injury. These findings identify CNP–orientin as a promising nanotherapeutic strategy for hypoxia-associated lung diseases and support further translational investigations.

## 1. Introduction

Pulmonary microcirculation is highly sensitive to fluctuations in oxygen tension. A reduction in alveolar oxygen rapidly triggers hypoxic pulmonary vasoconstriction (HPV), an adaptive mechanism that preserves ventilation–perfusion matching during acute hypoxia [1]. Although protective in the short term, sustained hypoxia gradually becomes pathological. Prolonged oxygen deprivation contributes to endothelial dysfunction, increased oxidative stress, and enhanced production of proinflammatory mediators [2]. Key features of this maladaptive response include macrophage and neutrophil infiltration, lipid peroxidation, impaired nitric oxide signaling, and depletion of antioxidant defenses [3].

Oxidative stress and NF-κB-mediated inflammatory signaling play central roles in the pathogenesis of acute lung injury (ALI). Studies by Zhang et al. have shown that impaired Nrf2/HO-1 signaling and activation of proinflammatory pathways contribute to lung tissue damage, highlighting potential therapeutic targets in ALI/ARDS [4].

Recent studies have also shown that the pulmonary microvasculature exhibits heterogeneous responses to hypoxia [5,6]. The magnitude and behavior of HPV depend not only on alveolar oxygen tension but also on intrinsic vascular oxygen-sensing pathways, ion channel regulation, redox-sensitive signaling networks, and the surrounding inflammatory microenvironment. Within this complex interplay, oxidative stress and inflammation mutually reinforce each other, accelerating acute tissue injury and early structural remodelling [1]. In rats, short-term hypoxia induces a rapid HPV response along with reproducible patterns of cytokine activation and oxidative stress [7].

Natural flavonoids, particularly Orientin, have gained attention for their strong antioxidant and anti-inflammatory properties. Orientin suppresses pro-inflammatory cytokines, reduces lipid peroxidation, and supports endothelial integrity [8]. Both in vitro and in vivo studies have further demonstrated that Orientin reduces reactive oxygen species and inflammatory responses [9]. More recent evidence has shown that Orientin attenuates inflammatory responses by inhibiting NF-κB signaling through activation of the Nrf2/HO-1 axis, further supporting its role as a regulator of cytokine-driven tissue injury [10]. However, the pharmacokinetic behavior of Orientin remains incompletely characterized, with previous studies reporting limited systemic exposure and rapid distribution and elimination [11,12].

Chitosan nanoparticles (CNPs), due to their biocompatibility, biodegradability, and capacity to enhance drug stability and cellular uptake, represent a promising strategy to improve the pharmacokinetic profile and biological activity of Orientin [13].

Experimental studies have shown that chitosan nanoparticles can modulate oxidative stress by reducing MDA and enhancing antioxidant defenses, including SOD and GSH, with reported involvement of the Nrf2/HO-1 pathway [14,15]. CNPs have also been reported to modulate inflammatory mediators, including NF-κB, iNOS, IL-1β, and TNF-α, as well as apoptosis-related pathways. These previously reported effects provided the rationale for evaluating oxidative stress, inflammatory signaling, and apoptosis-related parameters in the present study.

This study investigates the therapeutic potential of Orientin delivered with CNPs in a rat model of acute hypoxia-induced pulmonary inflammation. These findings are expected to support the development of new strategies for preventing or mitigating hypoxia-associated lung injury.

## 2. Materials and Methods

### 2.1. Study Design

In this study, 6-month-old female Sprague Dawley rats weighing 250–300 g (*n* = 48) were used. The rats were obtained from the Kafkas University Experimental Animal Unit, and this study was approved by the Kafkas University Local Ethics Committee for Animal Experiments (KAÜ-HADYEK) (approval No.: KAÜ-HADYEK/2024-224, date: 30 December 2024).

Throughout the experiment, rats were housed in a controlled environment at a temperature of 20–24 °C, 60% humidity, and a 12 h light/12 h dark cycle. Water and standard rat chow were provided ad libitum. During the one-week acclimatization period prior to this study, all rats were examined by a veterinarian and confirmed to be clinically healthy. All procedures were conducted in accordance with the ARRIVE guidelines and NIH Guide for the Care and Use of Laboratory Animals [16].

At the end of the experimental protocol, rats were anesthetized with a ketamine–xylazine combination consisting of ketamine hydrochloride (75 mg/kg) and xylazine (15 mg/kg), administered intramuscularly, in accordance with the approved animal ethics protocol. Adequate depth of anaesthesia was confirmed by the absence of corneal and pedal withdrawal reflexes. Terminal blood samples were collected under deep anaesthesia. The animals were then euthanized by cervical dislocation while under deep anaesthesia. Death was confirmed before tissue collection, after which lung tissues were harvested for subsequent biochemical, molecular, histopathological, and immunohistochemical analyses.

### 2.2. Grouping and Randomization

The sample size was determined by an a priori power analysis performed during the study design to ensure adequate statistical power while minimizing animal use. Based on this analysis, a total of 48 rats were randomly allocated to eight experimental groups (*n* = 6 per group), consisting of four normoxic and four hypoxic subgroups. The individual rat was considered the experimental unit.

(1) Normoxic Control (C): Maintained under normoxic conditions without any pharmacological intervention.
(2) Normoxic CNPs (CNP): Received chitosan nanoparticles under normoxic conditions.
(3) Normoxic Orientin (OR): Received orientin alone under normoxia.
(4) Normoxic CNPs + Orientin (CNP + OR): Treated with orientin-loaded chitosan nanoparticles under normoxic conditions.
(5) Hypoxia (H): Exposed to hypoxia protocol without any treatment.
(6) Hypoxia + CNPs (H + CNP): Exposed to hypoxia and administered chitosan nanoparticles.
(7) Hypoxia + Orientin (H + OR): Exposed to hypoxia and received orientin alone.
(8) Hypoxia + CNPs + Orientin (H + CNP + OR): Exposed to hypoxia and treated with orientin-loaded chitosan nanoparticles.

The rats were randomized using a computer-generated allocation sequence. During the 7-day hypoxia protocol, rats were kept in separate compartments to ensure uniform exposure. During the 7-day hypoxia period, treatments were administered according to group allocation; the normoxic control group received no intervention.

### 2.3. Establishment of the Hypoxic Environment

The experimental hypoxia model was adapted from previously established intermittent hypoxia protocols with minor modifications [5]. Rats were exposed to a hypoxic gas mixture (7% O_2_, 93% N_2_) for 8 h per day in an intermittent schedule designed to prevent severe physiological deterioration. This hypoxic cycle was repeated daily for 7 consecutive days.

Hypoxia exposure was carried out in a custom-built glove box constructed from 1-cm-thick transparent polypropylene, with gas-tightness ensured by triple-layer silicone seal. The chamber was equipped with 8″ neoprene gloves, a mixed gas regulator, and an exhaust valve. The temperature, humidity, and oxygen levels inside the chamber were continuously monitored using digital sensors. The gas mixture was delivered from 150-bar cylinders via a regulator system. To prevent hypercapnia and maintain stable hypoxic conditions, the gas flow rate was set at 20 L/min, considering increased respiratory rates and tidal volume changes under hypoxia. All manipulations were performed inside a glove box to avoid contact with normoxic air during the hypoxic period.

### 2.4. Preparation and Characterization of CNPs

Chitosan nanoparticles (CNPs) were synthesized using a previously reported method with slight modifications^18^. Briefly, chitosan (1 mg/mL) was dissolved in 1% acetic acid and stirred at 45 °C until a homogeneous solution was obtained. Subsequently, 75 mL of 0.1 N NaOH was added to 25 mL of the chitosan solution, followed by the addition of 20 mL of sodium citrate (Na_3_C_6_H_5_O_7_) under continuous stirring for 30 min. The resulting suspension was incubated at 95 °C for 24 h to promote nanoparticle stabilization. The synthesized CNPs were subsequently stored in the dark until further characterization and use.

For this study, CNPs were combined with Orientin to create CNPs/Orientin complexes. Orientin and CNP suspensions were mixed for 4–5 h using a magnetic stirrer to allow for electrostatic interactions between Orientin and the nanoparticles. The particle size and morphology were evaluated using Scanning Electron Microscopy (SEM; FEI QUANTA FEG 450, FEI Company, Hillsboro, OR, USA). Transmission Electron Microscopy (TEM; Hitachi High-Technologies Corporation, Tokyo, Japan) was also performed to further assess particle size distribution and morphological characteristics.

The hydrodynamic particle size, polydispersity index (PDI), and zeta potential of the CNPs and CNPs/Orientin formulations were determined using a Zetasizer Nano ZSP (Malvern Instruments Ltd., Malvern, Worcestershire, UK). Nanoparticle dispersions were prepared in ultrapure water, sonicated prior to analysis, and measurements were performed at 25 °C in triplicate. Encapsulation efficiency (EE) and drug loading (DL) of CNPs/Orientin were determined by an indirect method based on quantification of non-encapsulated orientin in the supernatant following centrifugation. Free orientin was quantified spectrophotometrically at 335 nm using a previously established calibration curve. The EE and DL values were subsequently calculated from the total amount of orientin initially added, the amount of free orientin, and the recovered nanoparticle mass. The experimental procedures for nanoparticle characterization and determination of EE and DL were performed in accordance with our previously established methodology [17].

For the in vitro release study, CNPs/Orientin were incubated in phosphate-buffered saline (PBS), and aliquots were collected at predetermined time points over 72 h, with an equivalent volume of prewarmed fresh PBS added after each sampling to maintain sink conditions. The concentration of released orientin was determined by UV–Vis spectrophotometry at 335 nm using the established calibration curve. Cumulative orientin release was calculated and expressed as a percentage of the total loaded amount. All experiments were conducted in triplicate, and the results were expressed as mean ± standard deviation. The release protocol was adapted from our previously published methodology [17].

### 2.5. Administration of Chitosan–Orientin Nanoparticles

Rats assigned to the CNP–Orientin treatment groups received 50 mg/kg/day of the CNP–Orientin formulation via intraperitoneal injection once daily throughout the 7-day experimental period. Treatments were administered during the hypoxia protocol to evaluate their protective effects against hypoxia-induced lung injury. To assess the early post-treatment inflammatory response, additional blood samples were collected 4–6 h after nanoparticle administration. The control group was subjected to the same sampling schedule but did not receive any treatment.

### 2.6. Blood and Tissue Sampling Procedures and Biochemical Analyses

Throughout the 7-day intermittent hypoxia exposure period, rats assigned to the treatment groups received once-daily intraperitoneal injections in accordance with their respective group assignments. The treatment regimens comprised orientin (40 mg/kg/day), chitosan nanoparticles (CNPs; 50 mg/kg/day), or orientin-loaded chitosan nanoparticles (Orientin-CNPs; 50 mg/kg/day). The 50 mg/kg/day dose refers to the total mass of the CNP–orientin formulation. Based on the experimentally determined drug loading of 11.6%, this formulation contained approximately 5.8 mg/kg/day of orientin. These doses were selected based on previously established experimental protocols and were not intended to provide an orientin-equivalent dose comparison between free orientin and the CNP–orientin formulation [17,18]. Treatments were administered consecutively for seven days under the same dosing schedule.

The effectiveness of the hypoxia protocol was verified by arterial blood gas analysis (PaO_2_) performed in randomly selected rats at the end of the first 8 h hypoxic exposure period. Mean PaO_2_ values ranged between 45–55 mmHg, confirming adequate hypoxic exposure. Hypoxia-related biological responses were evaluated at the end of the experimental protocol using tissue hypoxia markers.

Blood samples were obtained from the tail vein when required for protocol verification or by terminal cardiac puncture at the end of the experimental period.

### 2.7. Preparation of Lung Tissue Samples for Biochemical Analyses

Lung tissue samples allocated for biochemical analyses were homogenized in phosphate-buffered saline (PBS) at a tissue-to-buffer ratio of 1:9 (*w*/*v*). The homogenates were centrifuged at 10,000 rpm for 15 min, and the resulting supernatants were carefully collected and used for subsequent biochemical analyses.

### 2.8. Measurement of Lipid Peroxidation and Antioxidant Enzyme Activities

Lipid peroxidation in lung tissue was analyzed using a commercial ELISA kit. Malondialdehyde (MDA) levels in tissue supernatants were measured using ELISA kits (Cat. No.: E0156Ra, Bioassay Technology Laboratory [BT LAB], Shanghai, China) according to the manufacturer’s protocol. Superoxide dismutase (SOD) activity (Cat. No.: E0168Ra, BT-LAB) and reduced glutathione (GSH) levels (Cat. No.: E1101Ra, Bioassay Technology Laboratory [BT LAB], Shanghai, China) were determined using commercial ELISA kits (BT-LAB) according to the manufacturer’s protocols.

### 2.9. Measurement of Serum Cytokine Levels

Serum samples obtained from experimental groups were analyzed for inflammatory cytokines using commercial ELISA kits. Interleukin-1 beta (IL-1β) levels (Cat. No.: E0119Ra, Bioassay Technology Laboratory [BT LAB], Shanghai, China) and tumor necrosis factor-alpha (TNF-α) levels (Cat. No.: E0764Ra, Bioassay Technology Laboratory [BT LAB], Shanghai, China) were quantified to assess systemic inflammatory responses, following the manufacturers’ instructions.

### 2.10. Measurement of HIF-1α and iNOS Levels

Hypoxia-inducible factor-1 alpha (HIF-1α) and inducible nitric oxide synthase (iNOS) levels in lung tissue supernatants were determined using commercial rat-specific ELISA kits (HIF-1α:, Cat. No. E0210Ra; iNOS:, Cat. No. E0740Ra, Bioassay Technology Laboratory [BT LAB], Shanghai, China) according to the manufacturer’s instructions. All samples were analyzed in duplicate, and absorbance was measured at 450 nm using a microplate reader.

### 2.11. Radiological Assessment

To evaluate radiological alterations associated with hypoxia and treatment responses, thoracic imaging was performed in one randomly selected rat from each experimental group. Imaging was conducted at the end of the hypoxia protocol and immediately prior to euthanasia. The rats were lightly anesthetized to minimize motion artifacts, and thoracic imaging was obtained under standardized conditions. Radiological findings were assessed by an experienced radiologist for evidence of parenchymal changes, increased lung density, and hypoxia-related alterations. All radiological evaluations were performed blindly to ensure an objective assessment.

### 2.12. Histopathological and Immunohistochemical Analysis

Lung tissues were fixed in 10% neutral buffered formalin for 72 h. Following fixation, tissues were dehydrated through a graded ethanol series (50%, 70%, 80%, 96%, and 100%), cleared in xylene, and embedded in paraffin blocks.

Paraffin-embedded tissues were sectioned to a thickness of 5 µm using a rotary microtome (RM2125 RTS, Leica Biosystems Nussloch GmbH, Nussloch, Germany) and mounted onto glass slides. For histopathological evaluation, sections were stained using Mallory’s trichrome method as modified by Crossman. Stained sections were examined and photographed under a light microscope (Nikon Eclipse E600; Nikon Corporation, Tokyo, Japan) at 200× magnification.

Histopathological assessment of lung injury was performed using a semi-quantitative scoring system to evaluate pulmonary edema, vascular and alveolar structures, and bronchiolar pathology. Each parameter was graded on a scale of 0–3 (0 = normal, 1 = mild, 2 = moderate, 3 = severe), according to previously described criteria (Table 1) [19,20]. Pulmonary fibrosis was evaluated using the Modified Ashcroft Scoring System (Table 2) [21].

For immunohistochemical analysis, paraffin sections mounted on positively charged slides were incubated overnight at 37 °C. Sections were deparaffinized in xylene and rehydrated through graded ethanol series, followed by antigen retrieval in citrate buffer (pH 6.0) using microwave heating at 600 W (4 cycles of 5 min). After cooling, sections were washed with phosphate-buffered saline (PBS), and endogenous peroxidase activity was blocked using 3% hydrogen peroxide for 10 min in the dark. The protein blocking solution was then applied for 5 min.

Sections were incubated overnight at 4 °C with primary antibodies against NF-κB p65 (Cat. No. sc-8008; 1:100; Santa Cruz Biotechnology, Inc., Dallas, TX, USA) and Caspase-3 (Cat. No. sc-56053; 1:100; Santa Cruz Biotechnology, Inc., Dallas, TX, USA). After washing with PBS, sections were incubated with a secondary antibody and subsequently with horseradish peroxidase (HRP) for 30 min each. The detection system (Large Volume Detection System: anti-Polyvalent and HRP; Lab Vision, Thermo Fisher Scientific, Waltham, MA, USA) were used according to the manufacturer’s instructions.

Immunoreactivity was visualized using 3,3′-Diaminobenzidine (DAB) as the chromogen, followed by counterstaining with Mayer’s hematoxylin. Slides were mounted with Entellan and examined under a light microscope (Nikon Eclipse E600, Nikon Corporation, Tokyo, Japan).

For semi-quantitative evaluation, five randomly selected microscopic fields were analyzed per section. Staining intensity was scored as 0 (no staining), 1 (weak), 2 (moderate), and 3 (strong), while the percentage of positively stained cells was graded as 0 (0%), 1 (1–33%), 2 (34–66%), and 3 (≥66%) [22,23]. An overall immunoreactivity score was calculated based on these parameters. All histopathological and immunohistochemical evaluations were performed in a blinded manner by an experienced histologist.

### 2.13. Quantitative Real-Time PCR Analysis

Total RNA was isolated from lung tissue samples after homogenization in liquid nitrogen using a commercial RNA isolation kit (EcoTech Biotechnology, Erzurum, Turkey), according to the manufacturer’s instructions. RNA concentration and purity were assessed using a NanoDrop spectrophotometer (BioTek Instruments, Inc., Winooski, VT, USA), and samples with acceptable 260/280 nm ratios were used for further analyses.

Complementary DNA (cDNA) was synthesized from isolated RNA using a reverse transcription kit (EcoTech Biotechnology, Erzurum, Turkey) following the manufacturer’s protocol. Quantitative real-time PCR (qPCR) was conducted on a Rotor-Gene Q system (QIAGEN GmbH, Hilden, Germany) using SYBR Green Master Mix (EcoTech Biotechnology, Erzurum, Turkey).

Gene expression analysis included pro-apoptotic (*Caspase-3*) and anti-apoptotic (*Bcl-2*) markers, oxidative stress–related genes (*HO-1* and *NRF2*), and inflammatory markers (*NF-κB* and *TNF-α*). *β-actin* was used as the internal reference gene for normalization (Table 3). All reactions were performed in triplicates. Relative gene expression levels were calculated using the 2^−ΔΔCt^ method and expressed relative to the control group. Melt curve analysis was performed after each run to confirm the specificity of amplification.

The primary outcome measure was the attenuation of hypoxia-induced pulmonary injury, assessed by the histopathological lung injury score. Secondary outcome measures included oxidative stress markers (MDA, SOD, and GSH), inflammatory cytokines (TNF-α and IL-1β), HIF-1α and iNOS levels, immunohistochemical findings, and apoptosis-related gene expression.

### 2.14. Statistical Analysis

Statistical analyses were performed using SPSS software (version 25.0). The distribution of continuous variables was assessed using the Kolmogorov–Smirnov test. Normally distributed data are expressed as mean ± standard deviation. One-way ANOVA was used to compare three or more independent groups, followed by Tukey’s post hoc test for pairwise comparisons when an overall significant difference was detected. Non-normally distributed data are presented as median (minimum–maximum) and were analyzed using appropriate non-parametric tests. A *p* value < 0.05 was considered statistically significant.

## 3. Results

### 3.1. Characterization of CNPs and CNPs/Orientin

**Figure 1:**
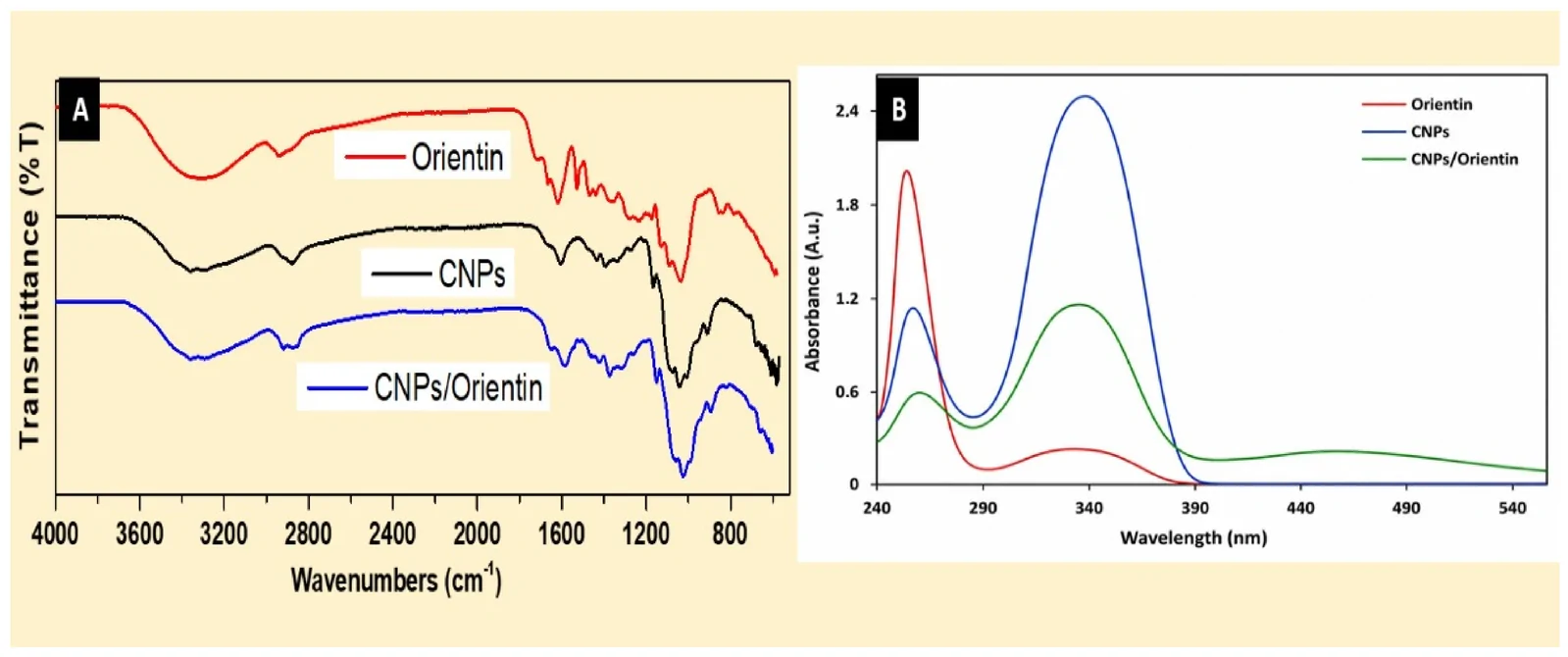
Spectroscopic characterization of free orientin, CNPs, and CNPs/Orientin. (**A**) FT-IR spectra and (**B**) UV–Vis absorption spectra of the Orientin, CNPs and CNPs/Orientin. CNPs: chitosan nanoparticles. (The FT-IR spectra were vertically offset).

The physicochemical characteristics of free orientin, blank chitosan nanoparticles (CNPs), and orientin-loaded chitosan nanoparticles (CNPs/Orientin) were comparatively evaluated by Fourier-transform infrared (FT-IR) and ultraviolet–visible (UV–Vis) spectroscopy (Figure 1). The comparison of the three spectral profiles provided complementary information regarding the characteristic functional groups of the individual components and the physicochemical changes associated with the incorporation of orientin into the chitosan nanoparticle matrix.

The FT-IR spectrum of free orientin exhibited a broad absorption band at approximately 3300 cm^−1^, which can be attributed primarily to O–H stretching vibrations, together with a band around 2900 cm^−1^ corresponding to C–H stretching vibrations. These spectral features are consistent with the hydroxyl-rich structure of orientin. In comparison, the spectrum of blank CNPs showed the characteristic vibrational bands of the chitosan matrix. A broad band centered at approximately 3350 cm^−1^ was observed, corresponding mainly to O–H/N–H stretching vibrations. The absorption band near 1630 cm^−1^ was associated with carbonyl-related vibrations, whereas the band around 1530 cm^−1^ could be attributed to N–H bending and C–N-related vibrations. The band at approximately 1375 cm^−1^ was associated with deformation vibrations of the chitosan backbone. Additional bands observed throughout the 1279–580 cm^−1^ region further reflected the characteristic vibrational fingerprint of chitosan (Figure 1A) [24].

Notably, the FT-IR profile of CNPs/Orientin differed from that of the blank CNPs while retaining the characteristic spectral features of the chitosan matrix (Figure 1A). The changes in the relative intensity and spectral profile of the characteristic bands following orientin incorporation suggest alterations in the chemical microenvironment of the nanoparticle matrix. These changes may be associated with intermolecular hydrogen bonding and other non-covalent interactions between the hydroxyl groups of orientin and the amino/hydroxyl groups of chitosan. Importantly, no prominent new absorption band indicative of the formation of a new covalent functional group was evident in the CNPs/Orientin spectrum. Therefore, the observed spectral modifications are more consistent with physical incorporation and/or intermolecular interactions of orientin within the chitosan nanoparticle matrix rather than the formation of a new covalent chemical structure. Collectively, the FT-IR findings support the successful incorporation of orientin into the CNP system while indicating preservation of the fundamental chitosan structure.

The UV–Vis spectra further demonstrated distinct spectral characteristics for free orientin, CNPs, and CNPs/Orientin (Figure 1B). Free orientin exhibited a prominent and sharp absorption maximum at approximately 255–260 nm, accompanied by a relatively weak broad absorption feature in the higher-wavelength region. In contrast, blank CNPs displayed a substantially different spectral profile, characterized by a broad and intense absorption band centered at approximately 330–340 nm. This broad absorption feature represents the characteristic optical response of the synthesized chitosan nanoparticle system. Following incorporation of orientin, the UV–Vis profile of CNPs/Orientin retained the broad absorption band in the ~330–340 nm region, although its intensity was markedly lower than that observed for the blank CNPs. The preservation of the broad CNP-associated absorption band together with the altered absorption intensity and spectral profile after orientin incorporation indicates that the optical environment of the nanoparticle system was modified following loading. The differences observed among the three formulations are particularly relevant when the spectra are considered together. The sharp absorption maximum of free orientin at approximately 255–260 nm was not retained as an isolated dominant peak in the CNPs/Orientin spectrum. Instead, the loaded formulation exhibited a spectral profile dominated by the broad absorption characteristics of the CNP matrix, accompanied by changes in absorption intensity and a weak extended absorption region at longer wavelengths. This behavior may reflect changes in the electronic microenvironment of orientin following its incorporation into the chitosan matrix, potentially arising from interactions between orientin and the amino and hydroxyl groups of chitosan [25].

**Figure 2:**
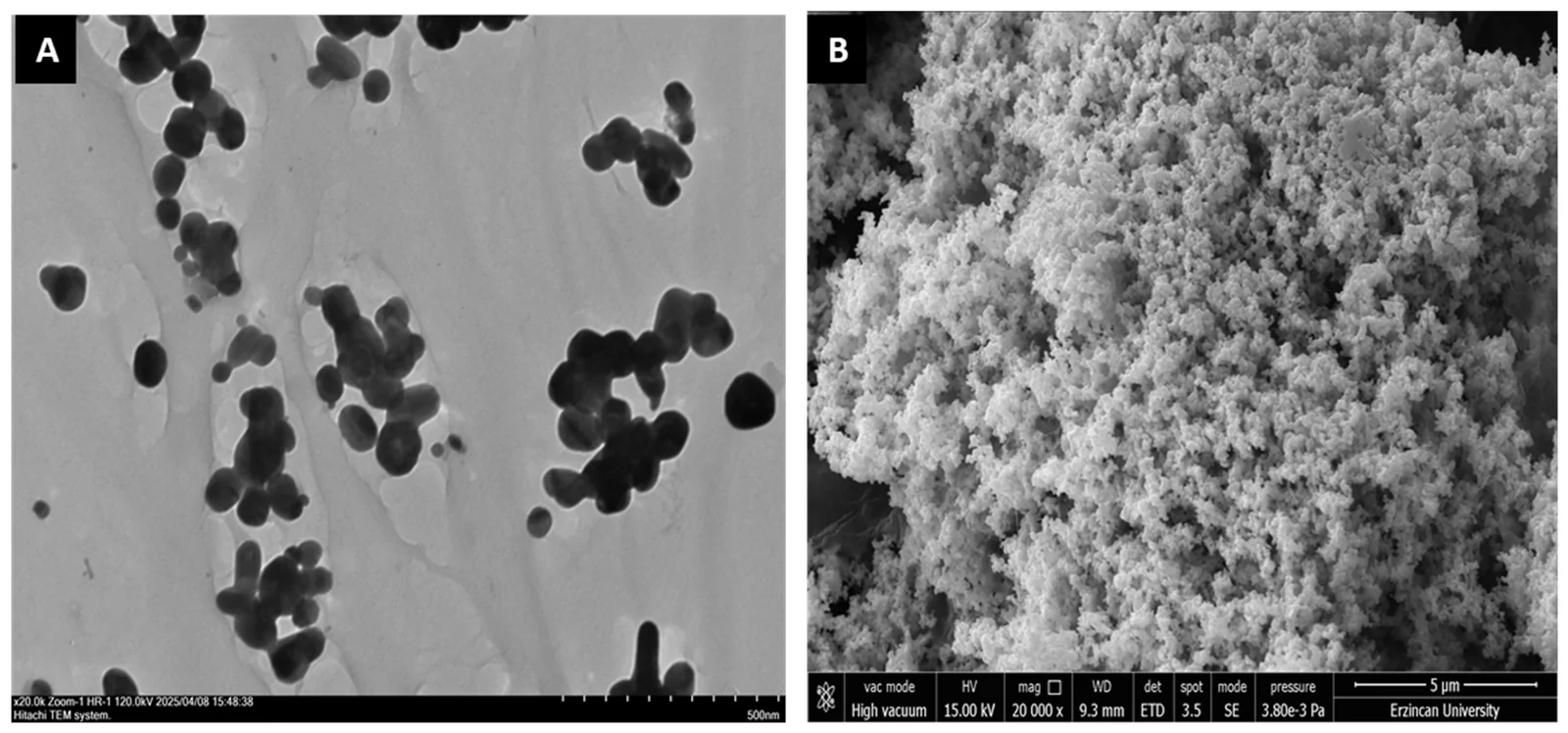
(**A**) The TEM spectrum of CNPs—(**B**) The SEM spectrum of CNPs. CNPs: chitosan nanoparticles; TEM: transmission electron microscopy; SEM: scanning electron microscopy.

Transmission electron microscopy (TEM) and scanning electron microscopy (SEM) were used to evaluate the morphology of the synthesized chitosan nanoparticles (CNPs) (Figure 2). TEM imaging (Figure 2A) revealed predominantly spherical to near-spherical nanoparticles with relatively uniform morphology, although some degree of particle aggregation was evident. The observed aggregation may be associated with interparticle interactions during sample preparation and drying. SEM analysis (Figure 2B) further demonstrated a highly aggregated, rough, and irregular surface morphology, with the nanoparticles forming compact clusters. The complementary TEM and SEM observations confirm the successful formation of nanoscale chitosan structures and indicate that the particles possess a tendency toward aggregation in the dried state. Overall, the morphological findings are consistent with the nanoparticulate characteristics of the synthesized CNPs and complement the physicochemical results obtained from spectroscopic and particle-size analyses [26].

The incorporation of orientin increased the mean particle size from 372.2 ± 3.3 nm for CNPs to 432.2 ± 0.9 nm for CNPs/Orientin, suggesting successful incorporation of orientin within or onto the chitosan nanoparticle matrix. The CNPs/Orientin formulation maintained a positive zeta potential of +27.3 ± 1.6 mV, although slightly lower than that of unloaded CNPs (+32.7 ± 1.9 mV), which may be attributed to the interaction of orientin with the positively charged chitosan surface. The PDI value of CNPs was 0.265 ± 0.008, indicating a relatively homogeneous particle-size distribution, whereas the incorporation of orientin resulted in a slight increase in PDI to 0.300 ± 0.004 for CNPs/orientin, while still maintaining a relatively homogeneous size distribution. Notably, CNPs/Orientin exhibited an encapsulation efficiency of 77.84 ± 1.7% and a drug-loading capacity of 11.6 ± 1.2%, demonstrating efficient incorporation of orientin into the nanoparticulate system (Table 4). Overall, the observed changes in particle size, surface charge, and PDI following orientin loading, together with the high encapsulation efficiency, support the successful formation of the sodium-citrate-based chitosan/orientin nanocarrier. These findings are consistent with previous reports showing that incorporation of active compounds into chitosan-based nanoparticles can alter particle size and surface characteristics while providing efficient encapsulation [25].

In Vitro Release Study: The in vitro release study demonstrated a sustained release profile of orientin from CNPs/Orientin over 72 h. Cumulative orientin release increased progressively from 7.4 ± 1.5% at 0.5 h to 11.3 ± 1.1% at 1 h, 18.2 ± 1.3% at 3 h, 30.7 ± 1.8% at 6 h, 41.4 ± 1.9% at 12 h, 56.2 ± 1.9% at 24 h, and 69.8 ± 2.1% at 48 h, reaching 77.2 ± 2.4% at 72 h. The gradual release pattern and absence of a pronounced initial burst suggest effective incorporation of orientin within the chitosan matrix and controlled diffusion from the nanoparticle network. These findings are consistent with the sustained-release behavior previously reported for chitosan nanoparticle-mediated orientin delivery [17]. Collectively, these findings support the potential of CNPs/Orientin as a controlled and sustained delivery platform for orientin.

### 3.2. Effects of Hypoxia and Treatments on HIF-1α and iNOS Levels

**Figure 3:**
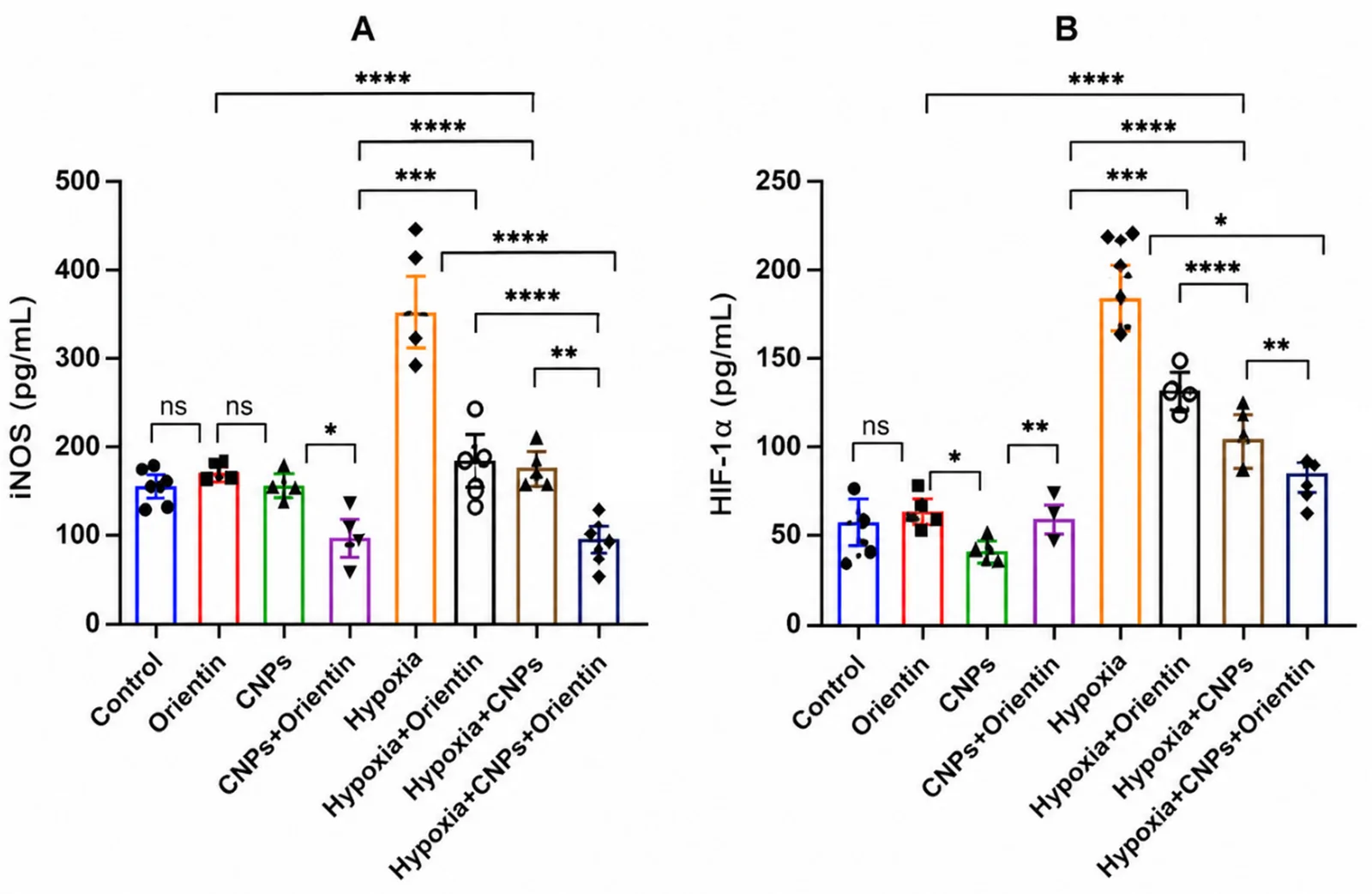
(**A**) Lung Tissue Level of Inducible nitric oxide synthase (iNOS) and (**B**) hypoxia-inducible factor-1 alpha (HIF-1α). (Data are presented as mean ± SD (*n* = 6). Statistical analysis was performed using one-way ANOVA followed by Tukey’s post hoc test. Statistical significance between the indicated groups is shown as ns (not significant), *p* > 0.05; * *p* ≤ 0.05; ** *p* ≤ 0.01; *** *p* ≤ 0.001; and **** *p* ≤ 0.0001).

To evaluate the hypoxic response and associated nitrosative/inflammatory signaling in lung tissue, HIF-1α and iNOS levels were measured. Exposure to hypoxia resulted in a significant increase in HIF-1α levels in lung tissue compared with the normoxic control group (*p* < 0.001). Treatment with Orientin, CNPs, or CNP–Orientin significantly attenuated the hypoxia-induced increase in HIF-1α levels (*p* < 0.05). However, no statistically significant differences in HIF-1α levels were observed between the CNP–Orientin treatment and the corresponding CNP or Orientin treatments under hypoxic conditions, indicating no additional effect of the combined formulation on this parameter. In addition, Orientin altered HIF-1α levels under normoxic conditions, and this finding is addressed in the Discussion. Similarly, iNOS levels were significantly elevated in the hypoxia group compared with the normoxic control group (*p* < 0.01). Treatment with Orientin, CNPs, or CNP–Orientin significantly reduced iNOS levels compared with the untreated hypoxia group (*p* < 0.05) (Figure 3).

### 3.3. Radiological Findings

**Figure 4:**
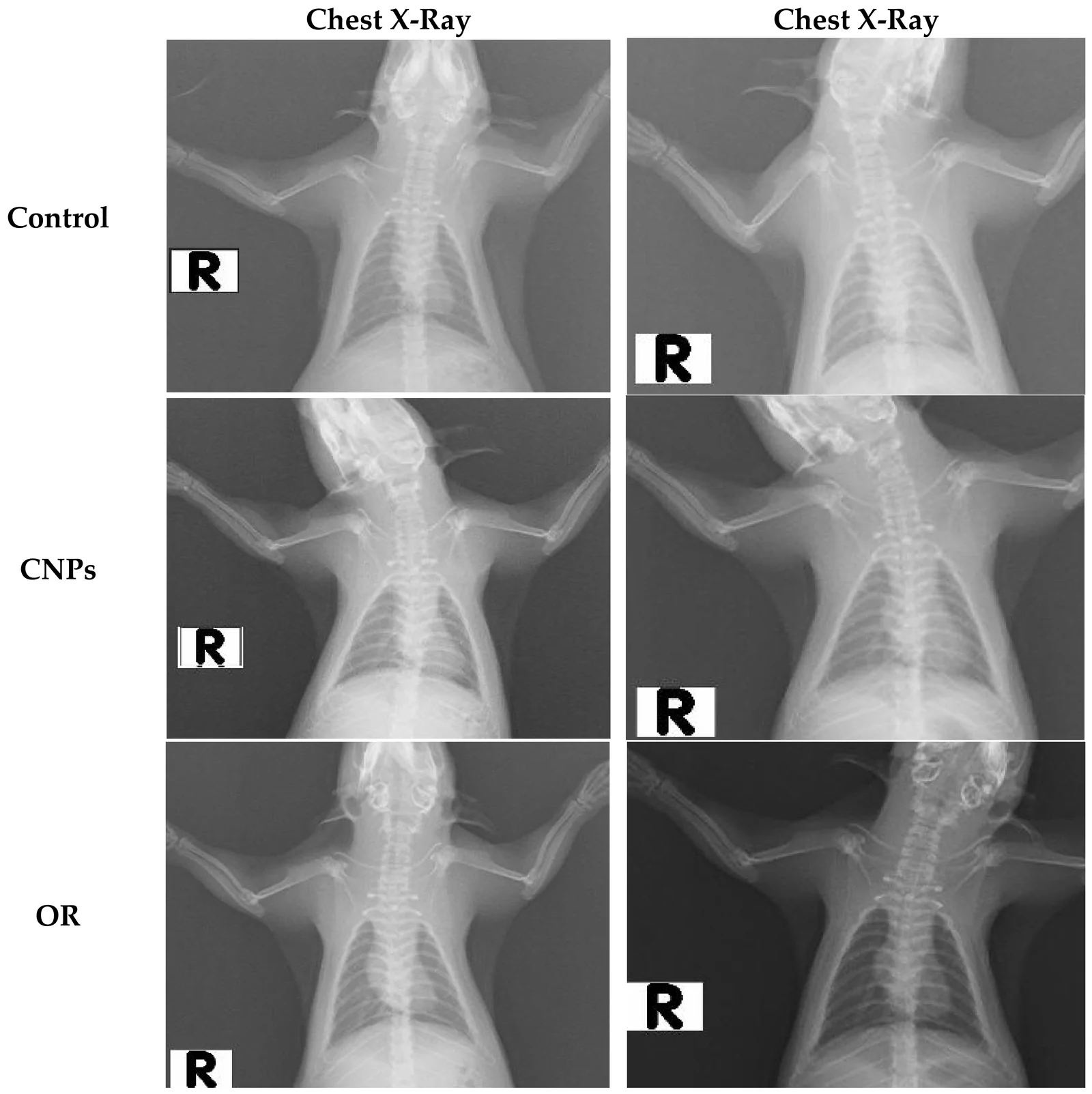

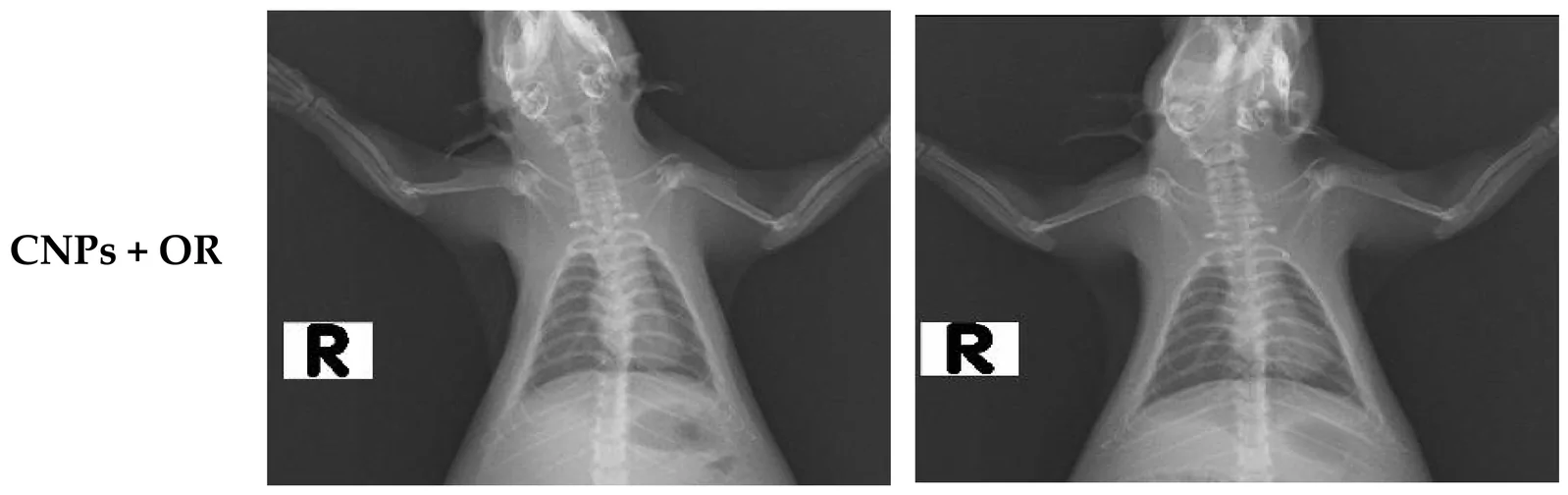
Representative chest X-ray images of experimental groups under normoxic and hypoxic conditions. The left column represents the normoxic groups, whereas the right column represents the corresponding hypoxic groups. CNPs: chitosan nanoparticles; OR: Orientin; CNPs + OR: CNP–Orientin formulation; R: right-side marker. (CNPs: Chitosan nanoparticles, OR: Orientin, CNPs + OR: Orientin-loaded chitosan nanoparticles, R: Right side marker).

To assess whether hypoxia-induced pulmonary injury was accompanied by detectable structural changes in the lung, chest radiographs were evaluated across the experimental groups. Representative posteroanterior chest X-ray images obtained at the end of the experimental protocol demonstrated comparable lung aeration and parenchymal appearance in all normoxic groups (control, CNPs, OR, and CNPs + OR), with no evident radiological abnormalities. In contrast, rats exposed to hypoxia showed a mild but discernible increase in overall lung density, consistent with reduced aeration under hypoxic conditions. No marked focal consolidation, structural distortion, or other overt radiological abnormality was identified in any groups. Radiological assessments were performed in a blinded manner and are presented for qualitative comparison as supportive findings rather than primary outcome measures (Figure 4).

### 3.4. Histopathological Findings

To determine the structural consequences of hypoxia and the histological effects of the treatments, lung tissues were evaluated for alveolar, vascular, and inflammatory alterations. The histopathological injury scores were significantly higher in the hypoxia group than in the normoxic groups (control, CNPs, orientin, and CNPs + orientin), as indicated by the pairwise comparisons shown in the figure (*p* < 0.01–0.0001). No significant differences were observed among the normoxic groups (*p* > 0.05).

In hypoxia-treated groups, histopathological injury scores were lower following treatment with CNPs, Orientin, or CNP–Orientin compared with the untreated hypoxia group. Among these treatments, the CNP–Orientin group exhibited the lowest histopathological injury score, which was significantly lower than that of the untreated hypoxia group (*p* < 0.01).

Similarly, the Modified Ashcroft fibrosis scores were significantly increased in the hypoxia group compared with the normoxic groups, as indicated by the pairwise comparisons presented in the figure (*p* < 0.01–0.0001), whereas no significant differences were observed among the normoxic groups (*p* > 0.05).

**Figure 5:**
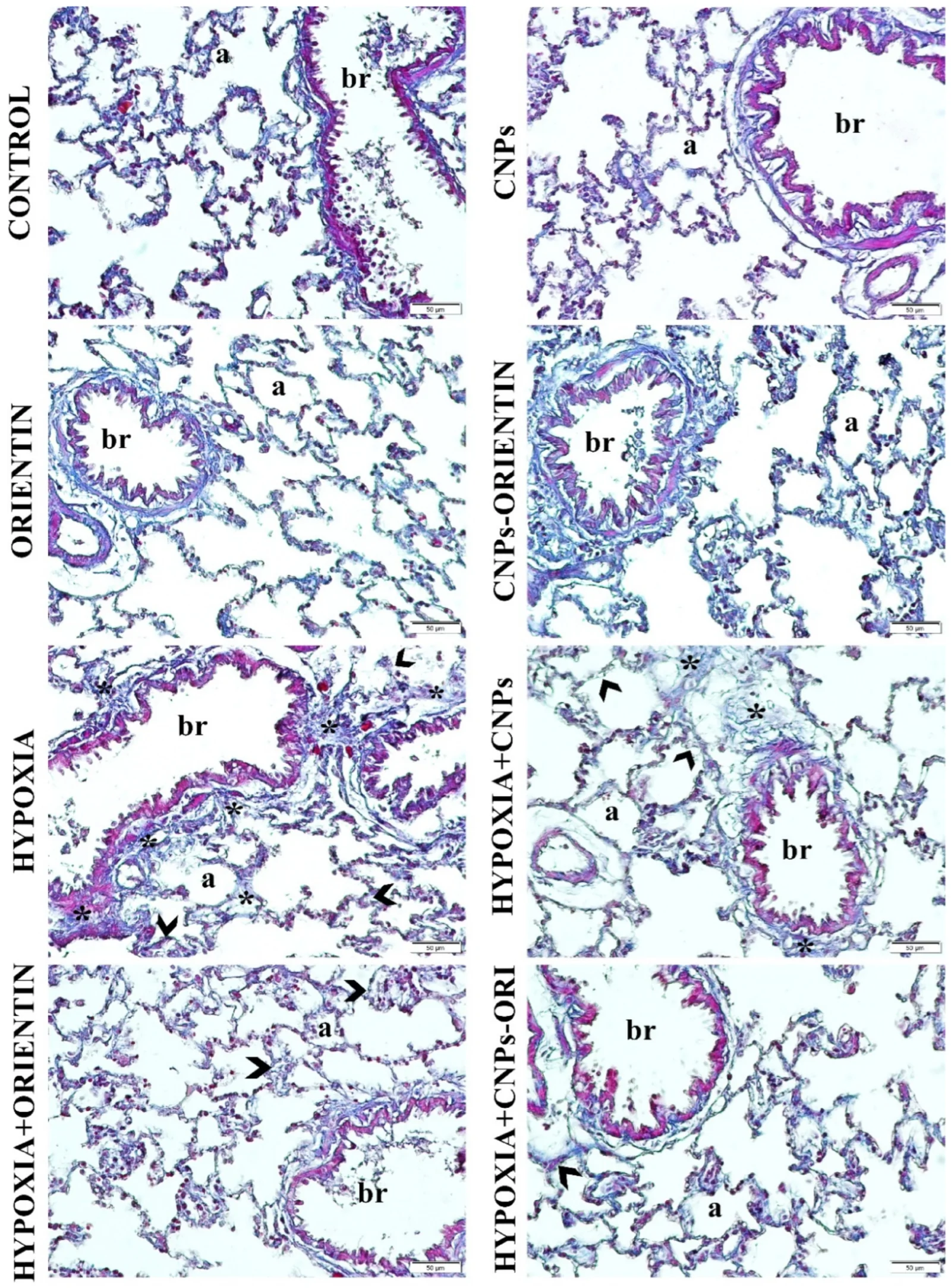
Representative micrographs of lung tissues from all experimental groups (Mallory’s trichrome staining as modified by Crossman, ×200 magnification). br: bronchiole; a: alveolus; arrow: degenerative alveolar changes and fibrosis; *: bronchiolar epithelial degeneration and fibrosis.

**Figure 6:**
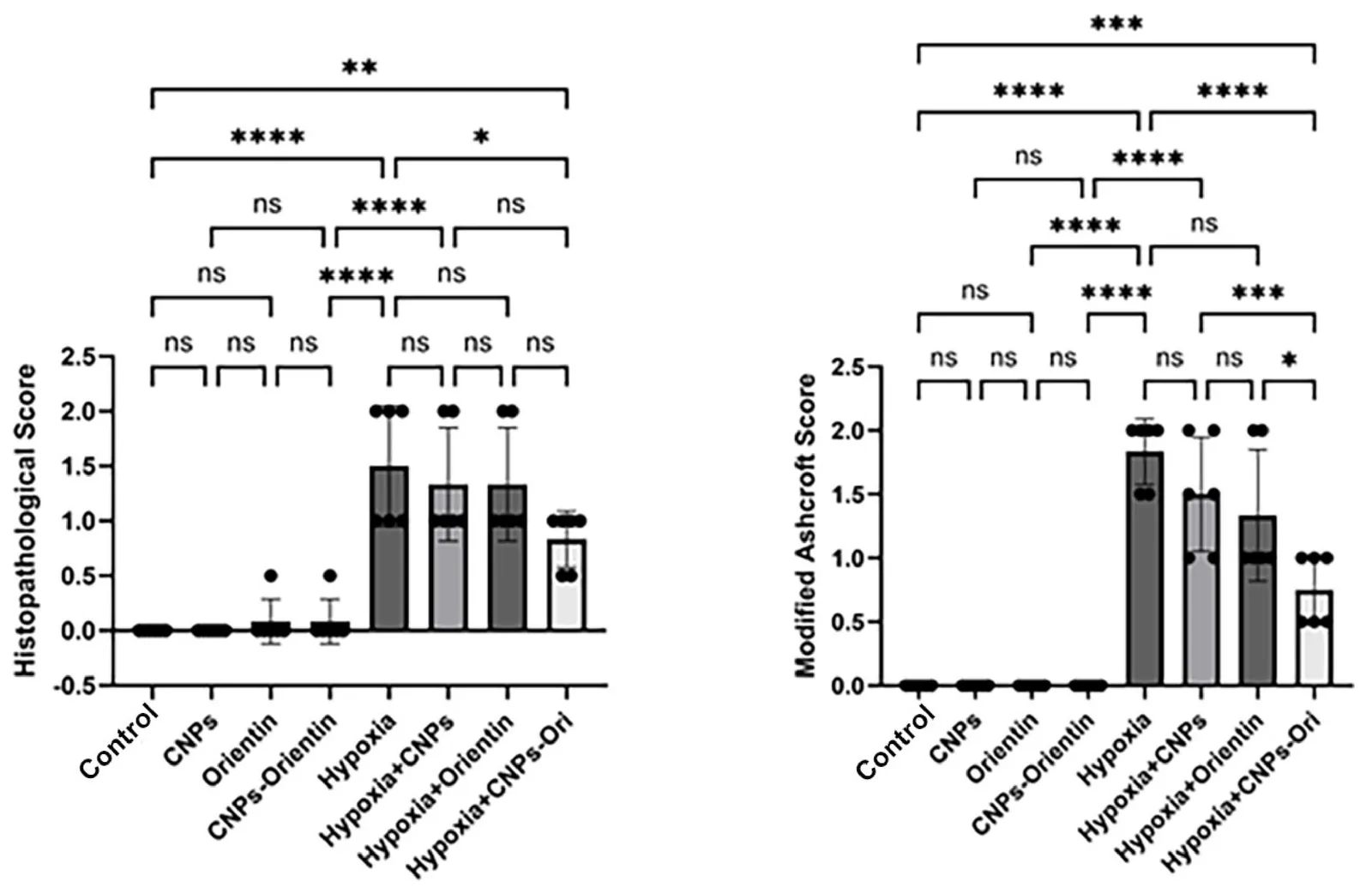
Quantitative evaluation of lung histopathological injury and pulmonary fibrosis in experimental groups. Semi-quantitative histopathological injury scores and Modified Ashcroft fibrosis scores are shown. Data are presented as mean ± SD (*n* = 6). Statistical analysis was performed using one-way ANOVA followed by Tukey’s post hoc test. Asterisks indicate statistically significant differences between groups (ns: not significant; * *p* < 0.05; ** *p* < 0.01; *** *p* < 0.001; **** *p* < 0.0001).

In hypoxia-treated groups, Modified Ashcroft fibrosis scores were reduced compared with the untreated hypoxia group. Among the treatment groups, the lowest Modified Ashcroft fibrosis score was observed in the CNP–Orientin group, which was significantly lower than that of the untreated hypoxia group (*p* < 0.001). Representative histopathological micrographs illustrating these findings are shown in Figure 5, while quantitative analyses of lung injury and fibrosis scores are presented in Figure 6.

### 3.5. Immunohistochemical Findings

To evaluate oxidative stress-, inflammation-, and apoptosis-related responses at the tissue level, immunohistochemical expression of Nrf2, HO-1, NF-κB, Caspase-3, and Bcl-2 was assessed. Immunohistochemical examination of lung tissues revealed significantly increased Caspase-3 scores in the hypoxia group compared to all normoxic groups (Control, CNPs, Orientin, and CNPs + Orientin) (*p* < 0.001–0.0001). No significant differences were observed among the normoxic groups (*p* > 0.05). In hypoxia-treated groups (Hypoxia + CNPs, Hypoxia + Orientin, and Hypoxia + CNPs + Orientin), Caspase-3 levels were lower than in the hypoxia group. Among these groups, the Hypoxia + CNPs + Orientin group showed the lowest Caspase-3 scores; however, this difference did not reach statistical significance compared with the hypoxia group.

**Figure 7:**
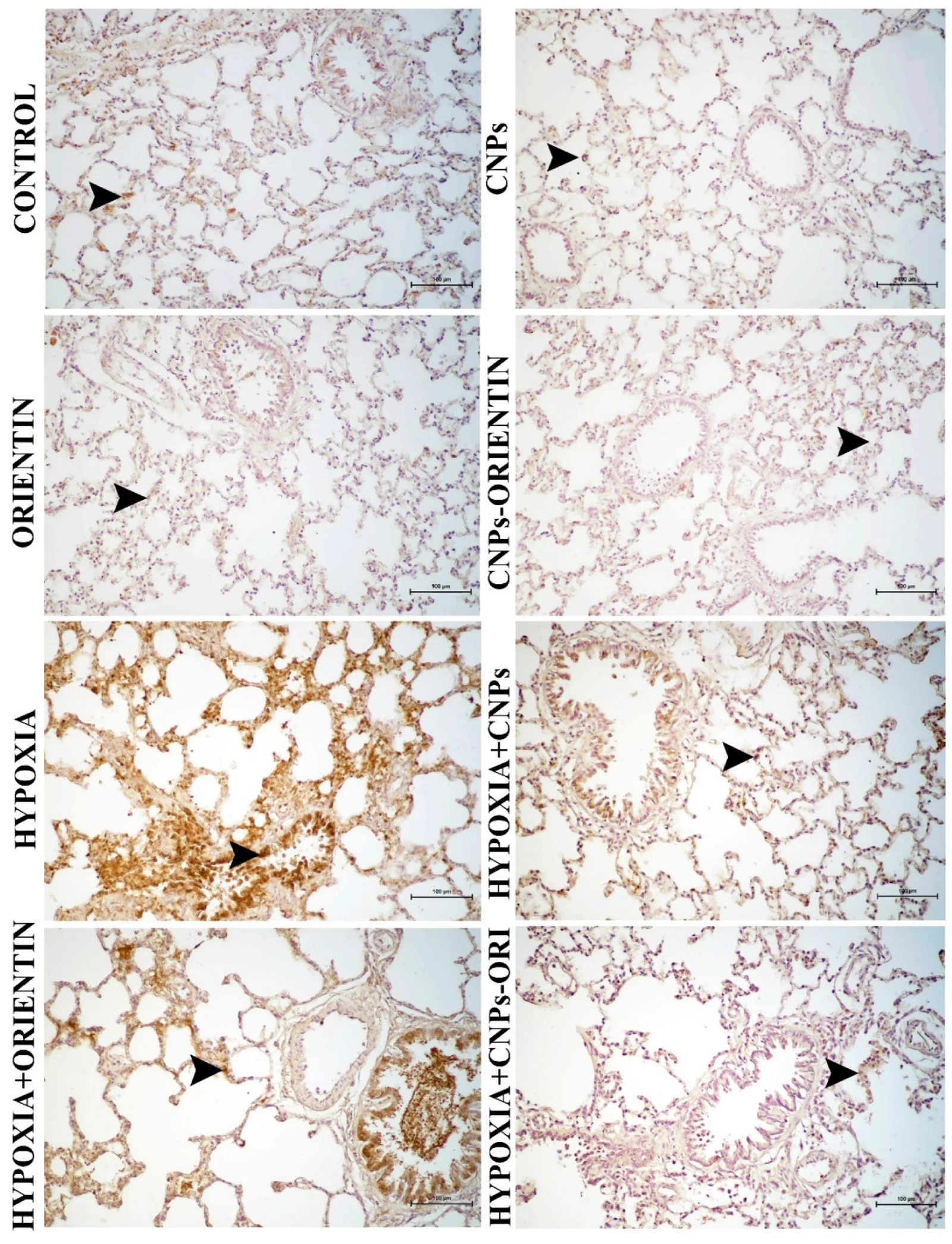
Representative immunohistochemical staining of Caspase-3 in lung tissues from all experimental groups. Arrows indicate immunoreactive cells. Staining was performed using the streptavidin–biotin peroxidase method. Images were captured at 200× magnification.

**Figure 8:**
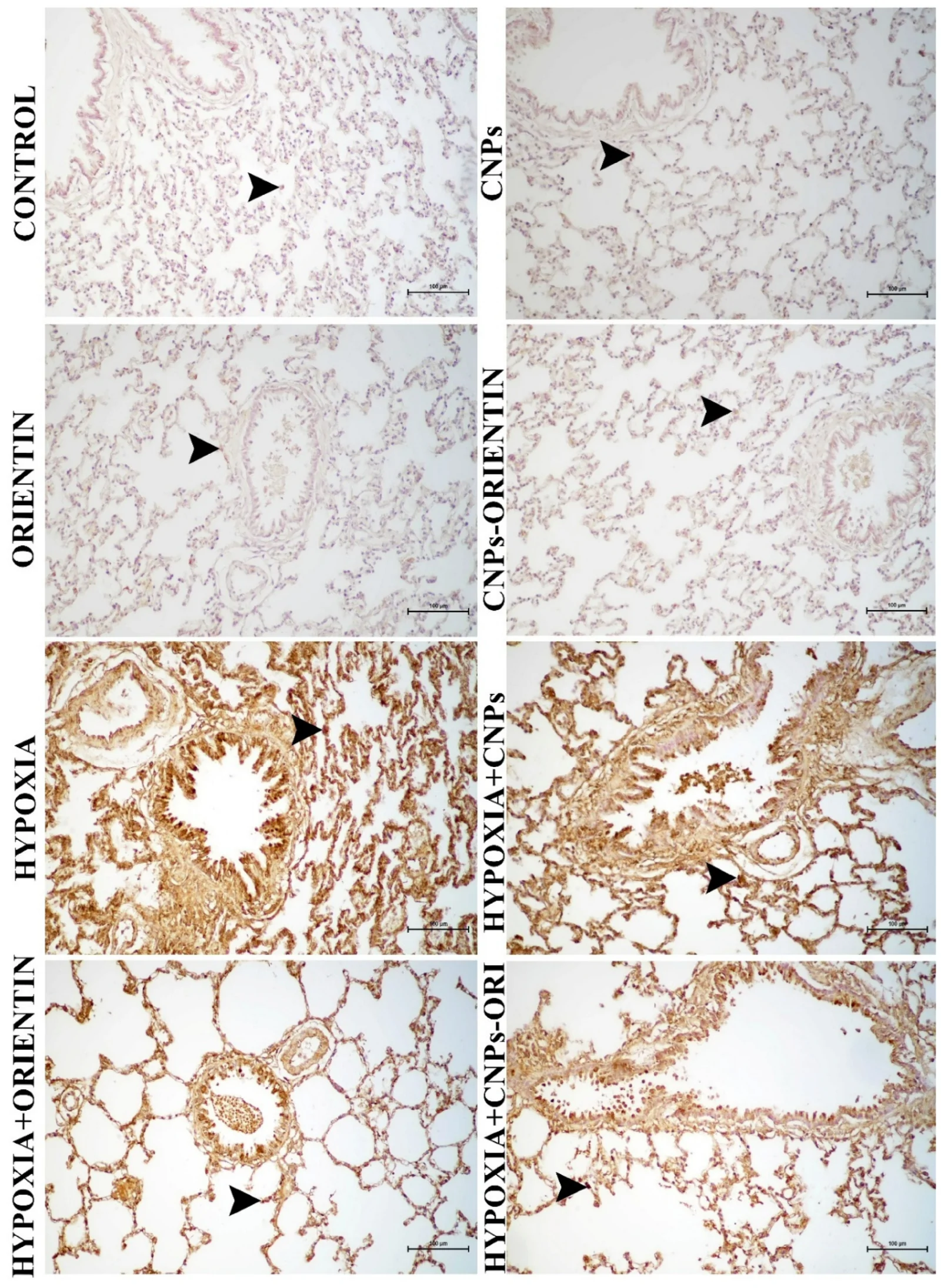
Representative immunohistochemical staining of NF-κB p65 in lung tissues from all experimental groups. Arrows indicate immunoreactive cells. Staining was performed using the streptavidin–biotin peroxidase method. Images were captured at 200× magnification.

**Figure 9:**
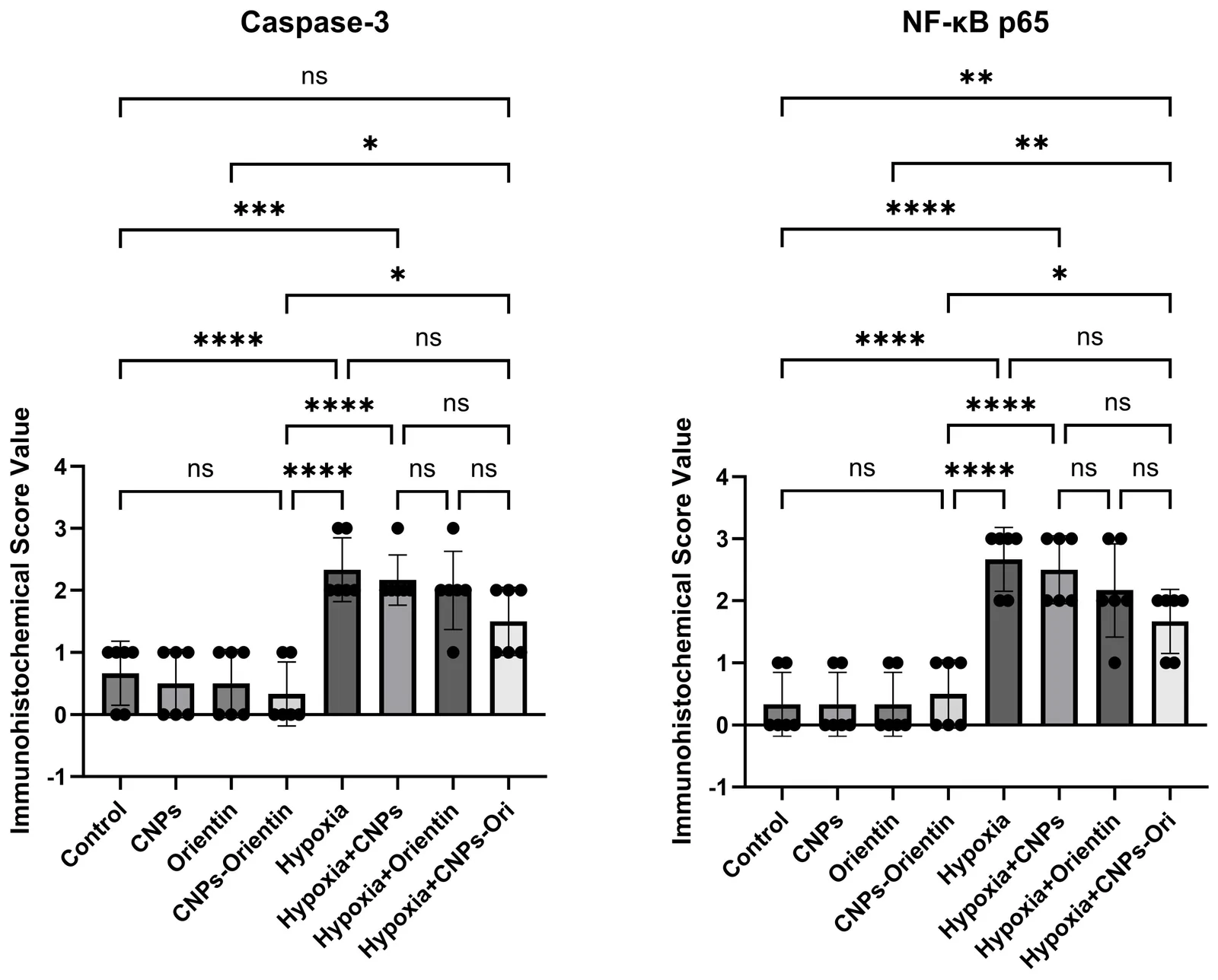
Quantitative immunohistochemical analysis of Caspase-3 and NF-κB p65 expression in lung tissues across all experimental groups. Data are presented as mean ± SD (*n* = 6). Statistical analysis was performed using one-way ANOVA followed by Tukey’s post hoc test. ns: not significant * *p* < 0.05, ** *p* < 0.01, *** *p* < 0.001, **** *p* < 0.0001.

NF-κB p65 immunohistochemical scores were significantly increased in the hypoxia group compared with all other groups (*p* < 0.01–0.0001). No significant differences were detected among the normoxic groups (*p* > 0.05). In the hypoxia-treated groups, NF-κB p65 levels were reduced compared to the hypoxia group. The Hypoxia + CNPs + Orientin group demonstrated the lowest NF-κB p65 scores; however, this reduction was not statistically significant compared with the hypoxia group. Representative immunohistochemical micrographs for Caspase-3 and NF-κB p65 are shown in Figure 7 and Figure 8, respectively, while quantitative analyses are presented in Figure 9.

### 3.6. Oxidative Stress Parameters

To assess the oxidative/antioxidant balance in lung tissue, SOD activity, MDA levels, and GSH levels were measured as indicators of antioxidant defence and lipid peroxidation. Lung tissue SOD activity differed significantly among groups (*p* < 0.05). The hypoxia group showed the lowest SOD levels compared to all normoxic groups (*p* < 0.05). SOD levels in the Hypoxia + CNPs group were significantly higher than in the hypoxic group (*p* < 0.05), while the Hypoxia + Orientin group showed intermediate values. No significant differences were observed among normoxic Control, CNPs, Orientin, and CNPs + Orientin groups (*p* > 0.05).

MDA levels were significantly higher in the hypoxic group than in the normoxic group (*p* < 0.05). MDA concentrations in the Hypoxia + CNPs and Hypoxia + CNPs + Orientin groups were significantly lower than in the hypoxic group (*p* < 0.05). MDA values in normoxic groups did not differ significantly from each other (*p* > 0.05).

**Figure 10:**
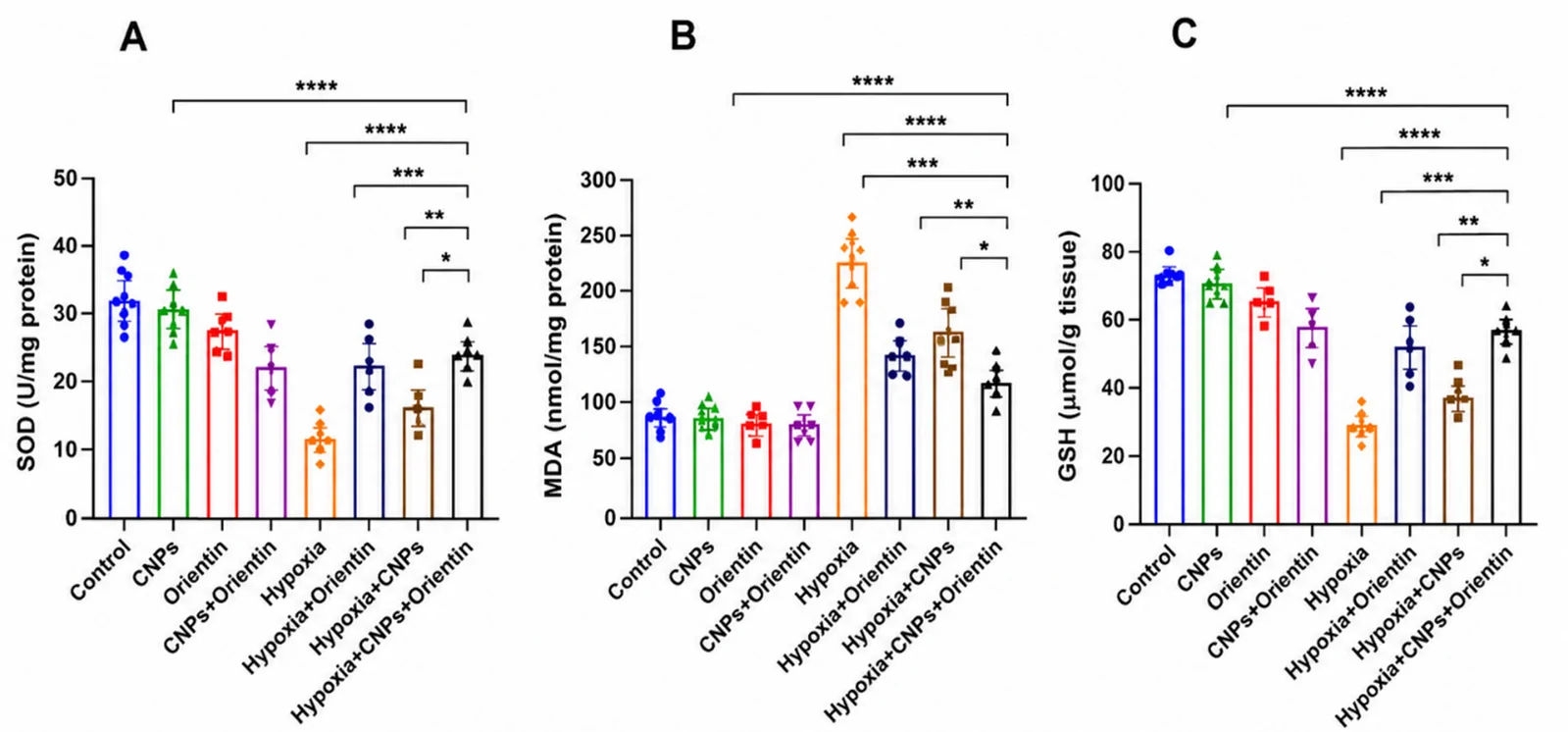
Effects of hypoxia and treatments on oxidative stress parameters in lung tissue. (**A**) Superoxide dismutase (SOD) activity, (**B**) malondialdehyde (MDA) levels, and (**C**) reduced glutathione (GSH) levels across the experimental groups. Data are presented as mean ± SD. Statistical analysis was performed using one-way ANOVA followed by Tukey’s post hoc test. Statistical significance between the indicated groups is shown as *p* > 0.05; * *p* ≤ 0.05; ** *p* ≤ 0.01; *** *p* ≤ 0.001; and **** *p* ≤ 0.0001.

GSH levels were significantly reduced in the hypoxia group compared with normoxic controls (*p* < 0.05). Treatment with CNPs significantly increased GSH levels in hypoxic rats (*p* < 0.05), whereas the Hypoxia + Orientin group showed partial restoration. No significant differences were observed among normoxic groups (*p* > 0.05) (Figure 10).

### 3.7. Serum Cytokine Levels

**Figure 11:**
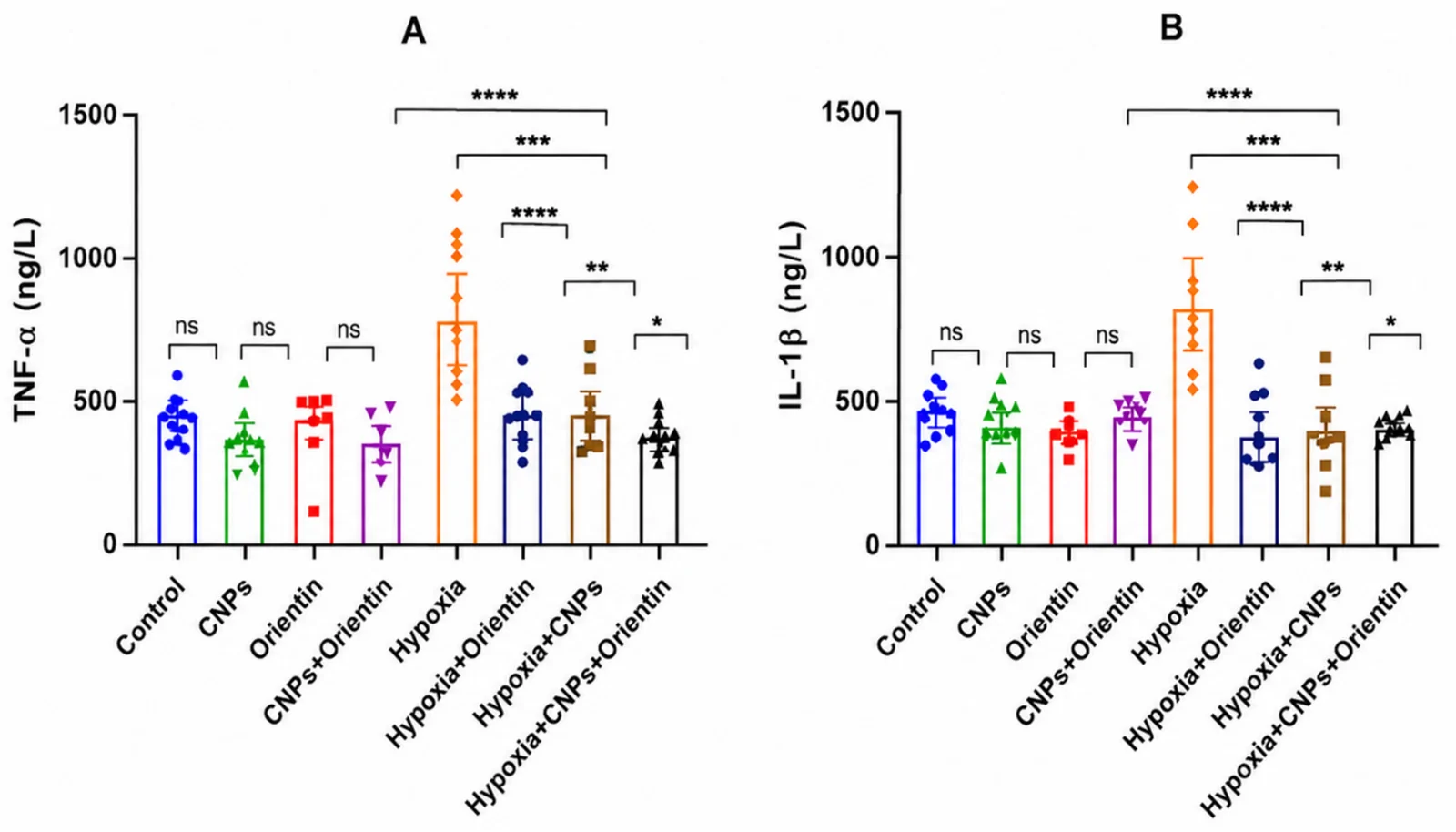
Effects of hypoxia and treatments on serum pro-inflammatory cytokine levels. (**A**) TNF-α and (**B**) IL-1β levels in the experimental groups. Data are presented as mean ± SD. Statistical analysis was performed using one-way ANOVA followed by Tukey’s post hoc test. Statistical significance between the indicated groups is shown as ns (not significant), *p* > 0.05; * *p* ≤ 0.05; ** *p* ≤ 0.01; *** *p* ≤ 0.001; and **** *p* ≤ 0.0001.

To evaluate the systemic inflammatory response associated with hypoxia and treatment, serum TNF-α and IL-1β levels were measured. TNF-α levels were significantly higher in the hypoxia group compared to all other groups (*p* < 0.05). According to post hoc analysis, the hypoxia group formed a distinct statistical group (letter b), whereas all normoxic groups and hypoxia-treated groups shared the same statistical grouping (letter a), indicating no significant differences among these groups. Similarly, IL-1β levels were significantly elevated in the hypoxia group compared with the remaining groups (*p* < 0.05). Post hoc comparisons showed that hypoxia-treated groups (Hypoxia + CNPs, Hypoxia + Orientin, and Hypoxia + CNPs + Orientin) did not differ significantly from normoxic groups and were assigned to the same statistical group (letter a) (Figure 11).

### 3.8. Quantitative Real-Time PCR Analysis Results

**Figure 12:**
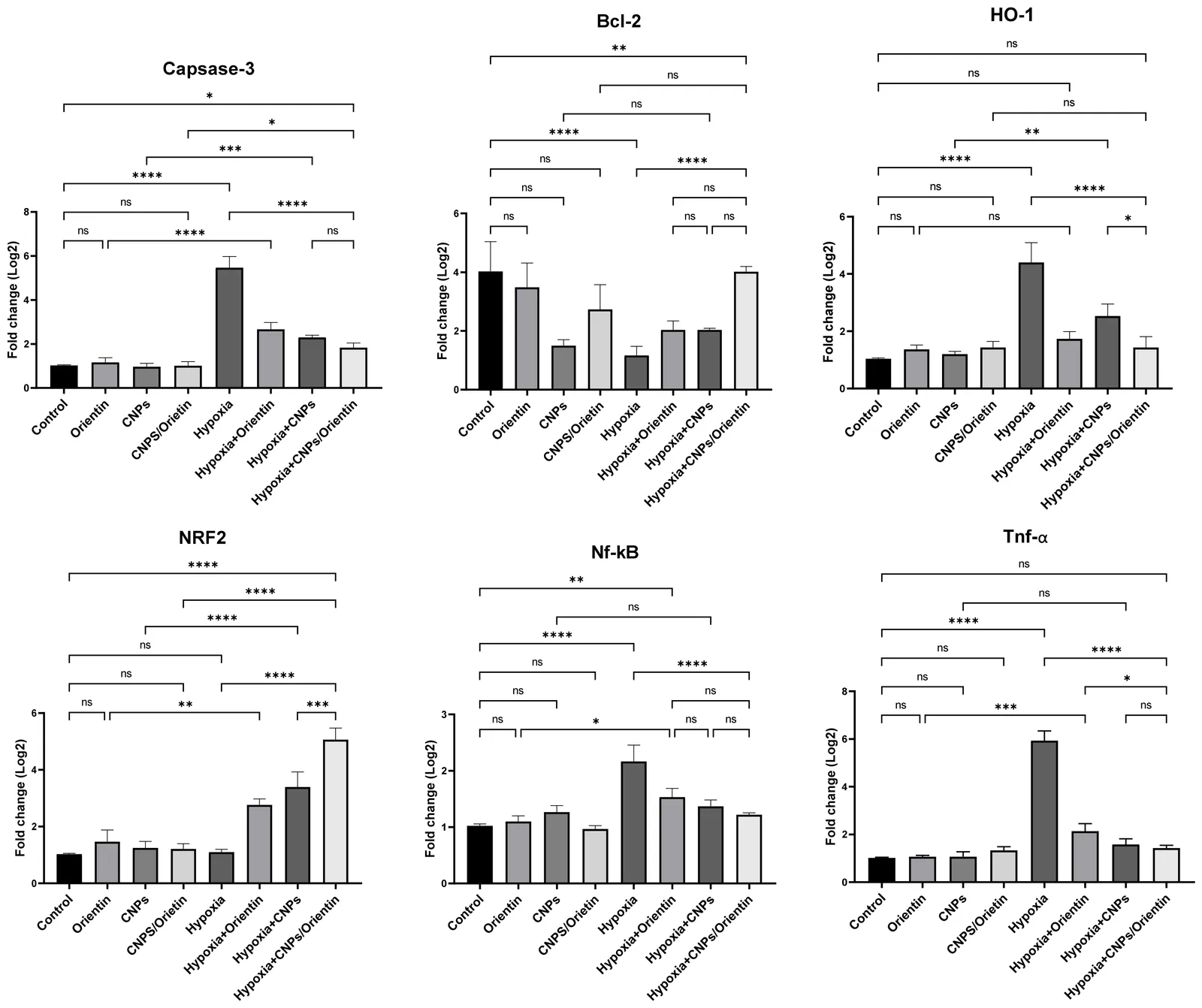
Relative mRNA expression levels of *Caspase-3*, *Bcl-2*, *HO-1*, *NRF2*, *NF-κB*, and *TNF-α* in rat lung tissue across experimental groups, determined by quantitative real-time PCR. Data are presented as mean ± SD (*n* = 6). Statistical analysis was performed using one-way ANOVA followed by Tukey’s multiple comparison test. * *p* < 0.05, ** *p* < 0.01, *** *p* < 0.001, **** *p* < 0.0001; ns, not significant.

To further characterize the molecular responses associated with oxidative stress, inflammation, and apoptosis, the expression levels of Nrf2, HO-1, NF-κB, Caspase-3, and Bcl-2 were evaluated by quantitative PCR. Quantitative RT-PCR analysis revealed significant alterations in the expression of apoptosis-, oxidative stress–, and inflammation-related genes across the experimental groups. Caspase-3 mRNA expression was significantly increased in the hypoxia group compared to the control and normoxic treatment groups (*p* < 0.05–0.0001), whereas caspase-3 levels were significantly decreased in the hypoxia-treated groups receiving Orientin, CNPs, or a combination thereof compared to the hypoxia-only groups (*p* < 0.05). Bcl-2 expression was also significantly decreased in the hypoxia group compared to the control group (*p* < 0.01–0.0001), whereas Bcl-2 expression was significantly higher in the hypoxia-treated groups than the hypoxia group (*p* < 0.05). HO-1 mRNA levels showed a marked increase in the hypoxia group compared with control (*p* < 0.0001); this increase was significantly attenuated in hypoxia-treated groups (*p* < 0.05–0.01). NRF2 expression was significantly upregulated in the hypoxia group relative to normoxic controls (*p* < 0.0001), with persistently elevated levels observed in hypoxia-treated groups, particularly in the Hypoxia + CNPs + Orientin group (*p* < 0.001). NF-κB expression was significantly higher in the hypoxia group compared with the control (*p* < 0.01–0.0001). Among hypoxia-treated groups, a significant reduction was observed in the Hypoxia + CNPs group compared to hypoxia alone (*p* < 0.05). TNF-α mRNA levels were significantly increased in the hypoxia group compared with the control and normoxic treatment groups (*p* < 0.001–0.0001). In contrast, TNF-α expression was reduced considerably in hypoxia-treated groups compared with hypoxia alone (*p* < 0.05), with no significant differences observed among treatment groups (Figure 12).

## 4. Discussion

This study demonstrates that hypoxia induces pronounced oxidative stress, inflammatory activation, and structural injury in lung tissue. Our findings show that treatment with Orientin-loaded chitosan nanoparticles (CNPs) significantly attenuates these pathological processes. The combined evaluation of biochemical, inflammatory, and histopathological parameters supports a substantial protective effect of the CNP–orientin formulation against hypoxia-induced lung injury. These results suggest that nanocarrier-based antioxidant strategies hold considerable potential for the prevention and treatment of hypoxia-related pulmonary injury.

To better understand the mechanisms underlying these protective effects, it is important to consider the key molecular pathways involved in hypoxia-induced lung injury. Hypoxia is a well-established trigger of pulmonary oxidative stress and inflammatory signaling [27]. Reduced oxygen availability disrupts mitochondrial respiration and promotes excessive production of reactive oxygen species, leading to lipid peroxidation, endothelial dysfunction, and activation of inflammatory cascades [28]. Consistent with recent experimental and clinical findings, hypoxia is associated with a significant increase in malondialdehyde (MDA) levels with reductions in endogenous antioxidant defenses such as superoxide dismutase (SOD) and glutathione (GSH) in lung tissue, indicating the development of pronounced oxidative stress and lipid peroxidation under hypoxic conditions [29,30].

Treatment with Orientin or CNPs alone resulted in partial improvement in oxidative stress parameters; however, these effects were insufficient to fully restore antioxidant balance. The CNP–Orientin formulation was also associated with attenuation of hypoxia-induced oxidative stress, including a significant reduction in MDA levels compared with untreated hypoxic rats. Interestingly, this biological activity was observed despite the formulation containing a markedly lower orientin-equivalent dose than the free-Orientin regimen. This observation may indicate efficient biological activity of the nanoparticle-associated formulation; however, dose-matched studies are required before conclusions regarding superiority can be drawn. Moreover, as the present study did not directly assess the bioavailability, pharmacokinetics, or tissue distribution of Orientin, the protective effects observed with the CNP–Orientin formulation should not be interpreted as direct evidence of improved bioavailability or pulmonary delivery. Previous studies have shown that chitosan-based nanoparticles can improve the solubility, cellular uptake, and tissue-level efficacy of polyphenols and flavonoids, leading to more pronounced antioxidant effects [13,31].

Similarly, Zhang et al. reported that nanoparticle formulations of flavonoids may produce stronger antioxidant responses than their free forms. Such effects have been attributed to altered stability, cellular interactions, or pharmacokinetic behavior of nanoparticle-associated compounds. However, because cellular uptake, pulmonary accumulation, and tissue retention were not directly evaluated in the present study, the greater biological responses observed with CNP–Orientin cannot be attributed to any specific delivery or biodistribution mechanism.

In contrast, several studies have shown that flavonoids can exert antioxidant effects even when administered alone; however, such effects are often observed at relatively high doses or within short-term experimental settings [31,32]. This may explain why Orientin administered alone produced only a limited antioxidant response in our study. These findings suggest that nanoparticle-based formulations may modify the biological effects of flavonoids; however, the underlying pharmacokinetic mechanisms require direct investigation. Similarly, previous studies by Erbas et al. have demonstrated the effectiveness of silver nanoparticles in improving biological activity [33,34].

In addition to oxidative stress, hypoxia was associated with a pronounced inflammatory response. In the present study, significantly elevated levels of IL-1β and TNF-α were observed in the hypoxia group, indicating activation of inflammatory pathways known to contribute to pulmonary tissue injury.

Our findings are further supported by Zhang et al., who reported that oxidative stress and NF-κB-mediated inflammatory signaling drive endothelial barrier disruption, glycocalyx damage, and increased vascular permeability in acute lung injury [35]. These cytokines are key mediators of endothelial dysfunction, leukocyte recruitment, and subsequent tissue damage during hypoxic stress [36]. Consistent with our findings, Lee et al. demonstrated that Orientin exerts potent anti-inflammatory effects by suppressing IL-1β, TNF-α production, NF-κB activation, and leukocyte–endothelial interactions, thereby preserving endothelial barrier integrity under inflammatory conditions [8].

The reductions in NF-κB, iNOS, and pro-inflammatory cytokines observed in the CNP–Orientin group are consistent with attenuation of hypoxia-associated inflammatory signaling. Nevertheless, these in vivo findings do not establish whether CNP–Orientin directly regulates these pathways within specific pulmonary cell populations. Hypoxia-induced activation of HIF-1α stimulates inflammatory pathways, including *NF-κB* signaling and *iNOS* expression, thereby amplifying vascular permeability and inflammatory cell infiltration. In the present study, hypoxia significantly increased HIF-1α and iNOS levels, whereas treatment attenuated these hypoxia-associated alterations. These findings suggest modulation of hypoxia-associated signaling and inflammatory responses; however, the present data do not establish whether these effects result from enhanced cellular delivery of Orientin. Interestingly, Orientin also altered HIF-1α levels under normoxic conditions, suggesting that its influence on *HIF-1α*-related signaling may not be restricted to hypoxic conditions. However, the biological significance and underlying mechanism of this finding cannot be determined from the present study and warrant further investigation. Under hypoxic conditions, although treatment was associated with lower HIF-1α levels compared with untreated hypoxic animals, CNP–Orientin did not produce a statistically significant additional effect compared with CNPs or Orientin alone. Thus, the present findings do not support a specific superiority of the CNP–Orientin formulation in regulating HIF-1α.

Histopathological evaluation yielded findings that closely paralleled the biochemical and molecular analyses. Lung tissues from hypoxic rats exhibited alveolar septal thickening, inflammatory cell infiltration, bronchiolar epithelial degeneration, and early fibrotic changes, as reflected by increased general injury and Modified Ashcroft scores. In contrast, rats treated with CNP–Orientin showed substantial preservation of alveolar architecture and significantly lower injury and fibrosis scores. The concordance between molecular, biochemical, and tissue-level findings strengthens the biological relevance of the observed therapeutic effects and indicates that molecular improvements translate into meaningful structural protection. Previous experimental studies have demonstrated that Ocimum sanctum, a plant rich in Orientin, confers significant tissue protection by attenuating oxidative damage, apoptosis, and structural injury, as evidenced by preserved tissue architecture and reduced inflammatory and degenerative changes [37].

Apoptosis-related gene expression analysis further supports the protective role of CNP–Orientin therapy. Hypoxia induced a significant increase in *Caspase-3* expression along with a reduction in *Bcl-2* expression, consistent with activation of mitochondrial apoptotic pathways under hypoxic stress [38]. Treatment with CNP–Orientin was associated with reduced *Caspase-3* expression and increased *Bcl-2* expression compared with untreated hypoxic animals, consistent with attenuation of apoptosis-related signaling in lung tissue. Similar anti-apoptotic effects of Orientin have been reported in experimental models, where Orientin or Orientin-rich plant extracts reduced caspase activation and preserved tissue architecture through antioxidant and mitochondria-protective mechanisms [39]. These findings suggest that CNP–Orientin treatment is associated with preservation of cellular integrity in lung tissue, together with attenuation of apoptotic, oxidative, and inflammatory injury.

The biological effects observed with the CNP–Orientin formulation may reflect multiple contributing factors. Chitosan nanoparticles have been reported to influence drug stability and biological activity, while chitosan itself may also exert antioxidant and anti-inflammatory effects [40]. However, the present study did not directly evaluate whether these factors altered Orientin bioavailability, tissue distribution, or pulmonary retention. Therefore, the mechanisms underlying the biological effects observed with the CNP–Orientin formulation remain to be clarified.

The intraperitoneal route used in the present study permits absorption of administered compounds from the peritoneal cavity into the systemic circulation, either directly or through lymphatic pathways, depending on their physicochemical characteristics [41]. Accordingly, systemic exposure following intraperitoneal administration may provide a plausible route through which CNP–Orientin could exert biological effects in the lung. Alternatively, intranasal or intratracheal administration may provide more direct pulmonary exposure. Chitosan-based nanoparticles have been investigated as pulmonary delivery systems because of their biocompatibility, mucoadhesive properties, and potential to improve local drug residence and controlled release [42]. Future studies directly comparing systemic and pulmonary routes, together with pharmacokinetic and biodistribution analyses, are therefore warranted to determine the optimal route of CNP–Orientin administration.

Despite these limitations, this study has several notable strengths. The use of a controlled hypoxia model combined with comprehensive evaluation of oxidative stress, inflammation, apoptosis, radiological findings, and histopathology provides an integrated assessment of the biological effects of the treatments. The concordant changes observed across multiple biological levels support the protective effects observed in the experimental model.

### Limitations

This study has several limitations. First, the biodistribution and pharmacokinetic profile of CNP–Orientin were not directly assessed, and Orientin concentrations in the lung or other major organs were not quantified. Therefore, the present study provides no direct evidence of enhanced bioavailability, preferential pulmonary accumulation, or increased tissue retention of Orientin. Future studies using labeled nanoparticles and quantitative tissue distribution analyses are required to address these aspects. Second, complementary in vitro experiments were not performed; therefore, the present study cannot determine whether the observed pulmonary effects of CNP–Orientin resulted from direct actions on lung parenchymal cells or from indirect systemic mechanisms. Future studies using hypoxic pulmonary epithelial cells, pulmonary microvascular endothelial cells, or macrophages should assess cell viability, intracellular ROS generation, cytokine secretion, cellular uptake, and pathway-specific protein expression to directly characterize the cellular mechanisms of CNP–Orientin. Third, the present study was not designed as a systemic toxicity study, and dedicated hepatic and renal safety parameters were not evaluated. Therefore, no definitive conclusions regarding the systemic safety of CNP–Orientin can be drawn from the present findings. An additional limitation is that the free-orientin and CNP–orientin groups were not matched for orientin-equivalent dose. The CNP–orientin formulation contained approximately 5.8 mg/kg/day of orientin, whereas free orientin was administered at 40 mg/kg/day. Therefore, direct dose-normalized efficacy comparisons between these groups cannot be made. Nevertheless, the substantial biological activity observed with the CNP–orientin formulation despite its lower orientin content is noteworthy and warrants further investigation in dose-matched studies. Finally, functional respiratory parameters, long-term outcomes, dose–response relationships, and chronic exposure conditions were not evaluated, and the experimental model does not fully reproduce the complexity of chronic hypoxic lung diseases in humans.

## 5. Conclusions

In conclusion, this study demonstrates that intermittent hypoxia induces significant oxidative, inflammatory, apoptotic, and structural lung injury, and that treatment with CNP–Orientin attenuates these pathological alterations in vivo. The observed protective effects were accompanied by favorable changes in oxidative stress-, inflammation-, and apoptosis-related markers in lung tissue. However, because complementary in vitro experiments were not performed, the present findings cannot distinguish direct cellular actions of CNP–Orientin from indirect systemic effects and should therefore be interpreted as pathway-associated responses rather than definitive evidence of direct cellular regulation. Further mechanistic studies using relevant pulmonary cell models are warranted to clarify the cellular mechanisms underlying these protective effects.

## Figures and Tables

**Table 1:** Histopathological scoring criteria for lung injury.

Score	Vascular Features	Extravascular and Alveolar Changes	Bronchiolar Features
0	Normal	Normal	Absent
1	Few erythrocytes in the interstitium	Mild inflammation	Mild inflammation
2	Vascular obstruction, moderate hemorrhage	Moderate inflammation, moderate septal thickening	Moderate inflammation, focal degeneration of bronchiolar wall
3	Severe hemorrhage and severe vascular occlusion	Severe inflammation, marked alveolar thickening	Severe degeneration of bronchiolar epithelium

**Table 2:** Modified Ashcroft Scoring System for Pulmonary Fibrosis.

Score	Description	Histological Features
0	Normal lung	Normal alveolar structure, no fibrosis in interstitial areas
1	Mild septal thickening	Minimal fibrosis with thin collagen fibers; alveolar structure preserved
2	More apparent fibrosis	Definite septal thickening, limited fibrotic areas; alveolar walls thickened
3	Mild fibrotic lesions	Localized fibrotic foci; surrounding alveolar structure preserved
4	Moderate fibrosis	Wider fibrotic areas; narrowing of alveolar spaces and structural distortion
5	Onset of diffuse fibrosis	Coalescing fibrotic areas; partial loss of alveolar spaces
6	Severe fibrosis	Extensive fibrosis with marked alveolar destruction; lung architecture severely distorted
7	Advanced fibrosis	Very widespread fibrotic areas with near-total loss of alveolar structure
8	Complete fibrotic obliteration	Entire lung parenchyma replaced by dense collagen; complete loss of alveolar spaces; honeycombing may be present

**Table 3:** Primer sequences used for gene expression analysis and gene functions.

Gene	Forward Primer (5′ → 3′)	Reverse Primer (5′ → 3′)	Function/Description
*Caspase-3*	GGT ATT GAG ACA GAC AGT GG	CAT GGG ATC TGT TTC TTT GC	Pro-apoptotic effector caspase; mediates the execution phase of apoptosis.
*Bcl-2*	CCTGTGGATGACTGAGTACCTG	AGCCAGGAGAAATCA AACAGAGG	Anti-apoptotic gene regulating mitochondrial integrity, protects cells from apoptosis.
*HO-1*	CTATCGTGCTGCGATGAAC	CAGCTCCTCAAACAGCTCAA	Stress-inducible cytoprotective enzyme; key regulator of oxidative response and heme degradation.
*NRF2*	CCCAGCACATCCAGACAGACA	GGCTGGGAATATCCAGGGCAA	Master antioxidant transcription factor; activates HO-1 and other cytoprotective genes.
*NF-κB*	CGTGAGGCTGTTTGGTTTGA	TCTGCCCTCCTGACTCTACT	Central regulator of inflammatory pathways; controls cytokine production and stress responses.
*TNF-α*	TGATCGGTCCAACAAGGA	TGCTGGTGGTTTGCTACGA	Pro-inflammatory cytokine; promotes inflammation and apoptosis.
*β-actin*	TCCTCCGTGAGCGAAAGTACTCT	GCTCGAAGTACAGTCCGCTAGAA	Housekeeping gene used as reference for normalization of mRNA expression.

**Table 4:** Physicochemical characteristics of CNPs and CNPs/Orientin, including particle size, zeta potential, polydispersity index (PDI), encapsulation efficiency (EE), and drug loading (DL).

Nanoparticle	Particle Size (nm)	Zeta Potential (mV)	PDI	Encapsulation Efficiency (%)	Drug Loading (%)
CNPs	372.2 ± 3.3	+32.7 ± 1.9	0.265 ± 0.008	—	—
CNPs/Orientin	432.2 ± 0.9	+27.3 ± 1.6	0.300 ± 0.004	77.84 ± 1.7	11.6 ± 1.2

Data are presented as mean ± SD.

## Data Availability

The original contributions presented in this study are included in this article. Further inquiries can be directed to the corresponding author.
